# Recent Progress in Single-Nucleotide Polymorphism Biosensors

**DOI:** 10.3390/bios13090864

**Published:** 2023-09-01

**Authors:** Kaimin Wu, Feizhi Kong, Jingjing Zhang, Ying Tang, Yao Chen, Long Chao, Libo Nie, Zhao Huang

**Affiliations:** Hunan Key Laboratory of Biomedical Nanomaterials and Devices, Hunan University of Technology, Zhuzhou 412007, China; 13682439859@163.com (K.W.); k15515385102@163.com (F.K.); ty3100403650@163.com (J.Z.); yingtang@hnu.edu.cn (Y.T.); chenyao717@hnu.edu.cn (Y.C.); chaolong4617@163.com (L.C.)

**Keywords:** biosensor, SNPs, fluorescence, electrochemistry, quartz crystal microbalance (QCM)

## Abstract

Single-nucleotide polymorphisms (SNPs), the most common form of genetic variation in the human genome, are the main cause of individual differences. Furthermore, such attractive genetic markers are emerging as important hallmarks in clinical diagnosis and treatment. A variety of destructive abnormalities, such as malignancy, cardiovascular disease, inherited metabolic disease, and autoimmune disease, are associated with single-nucleotide variants. Therefore, identification of SNPs is necessary for better understanding of the gene function and health of an individual. SNP detection with simple preparation and operational procedures, high affinity and specificity, and cost-effectiveness have been the key challenge for years. Although biosensing methods offer high specificity and sensitivity, as well, they suffer drawbacks, such as complicated designs, complicated optimization procedures, and the use of complicated chemistry designs and expensive reagents, as well as toxic chemical compounds, for signal detection and amplifications. This review aims to provide an overview on improvements for SNP biosensing based on fluorescent and electrochemical methods. Very recently, novel designs in each category have been presented in detail. Furthermore, detection limitations, advantages and disadvantages, and challenges have also been presented for each type.

## 1. Introduction

Single-nucleotide polymorphisms (SNPs) are the most common form of genetic variations in human genomes [1,2,3,4]. SNPs were first discovered in 1980; restriction endonuclease assays were used to determine the presence or absence of DNA cleavage sites in the past [5]. SNPs account for approximately 90 percent of the human genome, and a great deal of them were explored during the period of the Human Genome Project [6]. SNPs are highly universal in the human DNA polymorphisms, with a frequency of about 1 in 1000, and they have an abundance of 1% or more at the lowest frequency in the human population [6]. They have received considerable concern in all fields, such as drug toxicity, genetic variation, and human diseases, and SNPs are becoming important markers in clinical diagnostics and genetic research [7,8,9,10]. Therefore, the identification of SNPs is necessary for early clinical diagnosis, effective treatments, and better understanding of the gene function and health of an individual [8,11,12,13,14,15]. 

It has been reported that SNPs are frequently difficult to distinguish because of differences in only one base in the DNA sequence, corresponding to different alleles [16]. The specificity, sensitivity, and cost-effectiveness of method designs are decisive measures for improving SNP detection [17,18]. Moreover, it is crucial to exploit efficient assessment methods to detect SNPs in complicated genomes. The predominantly frequent SNP genotyping methods, including heteroduplex analysis, allele-specific oligonucleotide hybridization, enzymatic mismatch cleavage, oligonucleotide ligation, and single-strand conformation polymorphism analysis, were utilized for point mutation detection [19,20,21,22]. 

The main problem affecting hybridization stability is the specificity of hybridization, as the effect of individual base mispaired double-stranded overall stability is minimal [23]. The use of probes, such as binary DNA probes, molecular beacons (MBs), and DNA ligation forming probes, improves the stability and specificity of using structured probes compared to ordinary complementary strands [23,24]. However, many hybridization-based reactions cannot be multiply tested, confining the time of multiple sample interpretation [23].

In homogeneous hybridization reactions, TaqMan (TM) [25] or MB [26] probes are used for real-time polymerase chain reaction (RT-PCR) fluorescence detection for SNP genotyping. TM- and MB-based methods do not require PCR post-processing and label separation stages, which have the advantages of high throughput [27]. The high cost of machines and probes is the major disadvantage of this analytical method.

DNA microarrays are currently the most popular methods for SNP identification due to their combination of high throughput and cost-effectiveness [28]. However, they are limited by complicated procedures and lengthy operation time [29]. DNA Sequencing can distinguish SNPs accurately and rapidly [30]. However, the sequencing process is very expensive, especially when large numbers of samples are present. Expensive analytical instruments and sample specificity have been the key challenges for years [23,31,32]. Therefore, developing sensitive, rapid, and cost-effective methods to identify SNPs still remains a challenge. In recent years, a variety of biosensing and SNP genotyping techniques have been established. DNA biosensors offer great opportunities as an analytical tool for genetic screening and detection [33], which have high specificity and sensitivity over a broad spectrum of analytes [34]. Moreover, numerous SNPs in biosensors can be differentiated by producing microarrays, leading to reduced cost and large-scale detection. Many SNP biosensing strategies have been developed so far over the past decades. Optical, electrochemical, and piezoelectric methods are some of the most popular transduction techniques that have been used in fabricating SNP biosensing platforms [35,36]. In this review, we highlight the improvements of SNP biosensing platforms, focusing on fluorescent and electrochemical biosensors. The basic principles, detection sensitivity, and specificity of the developed biosensors are described, as well as their detection limitations, advantages/disadvantages, and challenges of biosensor-based technologies. In addition, it covers future trends in the field of sensing, indicating the enormous potential of SNP biosensing.

## 2. Biosensors for SNP Detection

DNA biosensors have been widely used in disease diagnosis, genetic variation, and SNPs due to their high sensitivity, fast response, simple operation, and low price in detecting specific sequence genes. For the past several years, various types of biosensors have been launched in this field and are divided into several types on the basis of their signal converter elements. The most common types of biosensors include fluorescent and electrochemical biosensors. 

### 2.1. Fluorescent Biosensors

As the most common type of optical biosensors, fluorescent biosensors are widely used in SNP detection due to their inherent simple operation and high sensitivity [37,38,39,40,41,42,43,44]. In fluorescent biosensors, when the fluorescent probes bind to the target substance, the signal is converted into a readable fluorescent signal by a transducer to achieve quantitative detection of a specific target.

Xiao et al. developed a novel branch-migration molecular probe (BM probe) that was capable of recognizing the existence of discovered or undiscovered single-base variations, including in highly GC-rich sequence regions up to 0.3–1% [45]. The introduction of the strand exchange and displacement reactions technique into oligodeoxynucleotide (ODN) molecular probes has resulted in an unprecedented level of improving the detection selectivity and specificity. Taking advantage of these features of toehold exchange (TE), Yu et al. developed a fluorescent assay based on toehold-mediated strand displacement and nuclease-mediated strand digestion for the detection of point mutations. This detection strategy exhibited a 50~1000-fold discrimination among all possible single-nucleotide mutations and a detection limit of 200 pM [46].

**Figure 1:**
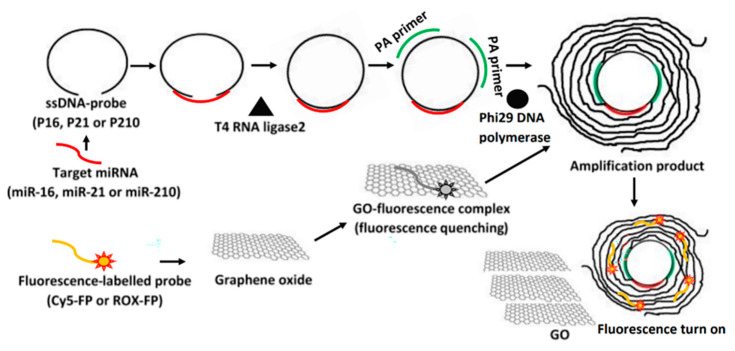
Schematic of fluorescence sensing detection strategy based on the multiple primers-mediated rolling circle amplification combined with a graphene oxide (MPRCA-GO) [53]. Copyright 2019 Royal Society of Chemistry.

Based on fluorescence quenching, graphene oxide (GO) has gained popularity and was sensitive enough to detect individual base mismatches [47,48,49,50]. Huang et al. exploited both the quenching efficacy and differential binding affinity of GO to overcome the temperature dependence distinguishment of single-base mutations in the allele-specific hybridization-based approach. The detection limit was 1.7 nM [51,52]. Furthermore, Krissana Khoothiam et al. used the superquenching properties of GO to efficiently perform fluorescence SNP detection (Figure 1). This strategy combined the designed ssDNA probe and T4 RNA ligase to effectively distinguish between perfectly matched and mismatched base pairs in DNA duplexes analyzed by multiple primers-mediated rolling circle amplification (MPRCA)-GO. The detection limit for this detection strategy was 0.87 fM [53]. In addition, carbon quantum dots (CQDs) have also been used to detect SNPs and are similar to GO based on fluorescence quenching, and they can be leveraged to develop a cost-effective test to detect SNPs in disease [54].

**Figure 2:**
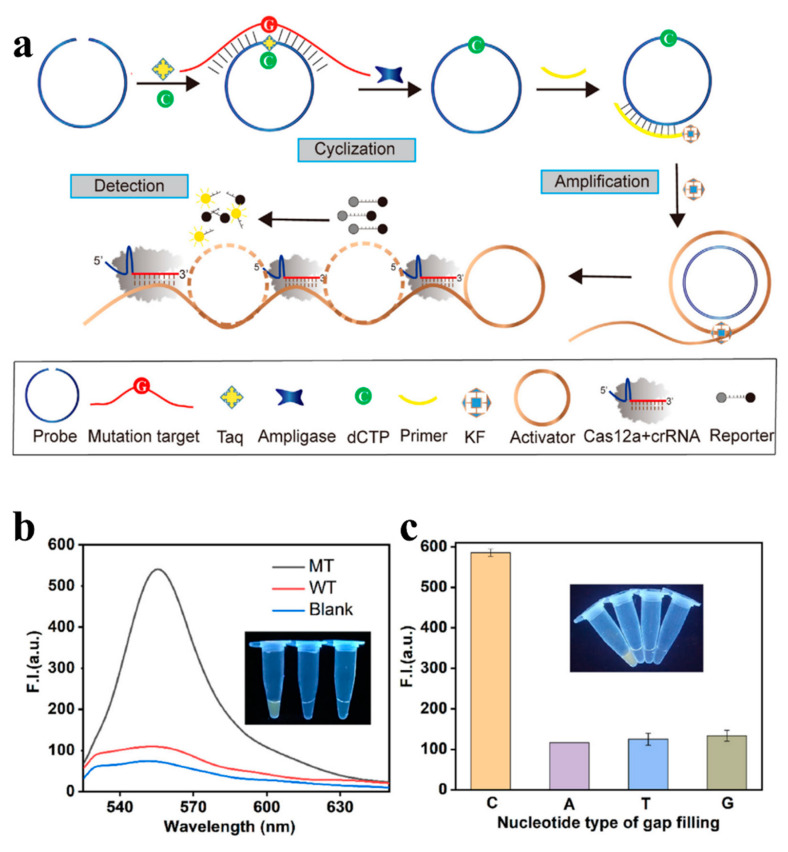
(**a**) Schematic explaining the method of the RCA-CRISPR/Cas12a technique for SNV detection; (**b**) representing the fluorescence intensity and real product photo corresponding to 200 nM mutant and wild-type targets and blank group; (**c**) showing the fluorescence intensity corresponding to the gap-filling with different nucleotides. Reproduced with permission from [56]. Copyright 2021 Elsevier.

To achieve higher SNP detection sensitivity, the biosensor is usually designed combined with some nucleic acid amplification technique. Rolling circle amplification (RCA) is a simple, but efficient, isothermal amplification technique that is commonly used to amplify short DNA primers to generate many long, linear, single-stranded DNA molecules with repetitive sequences, which are complementary to circular DNA templates [55]. Cao et al. constructed a fluorescence analytical method combining CRISPR/Cas12a and RCA techniques to detect single-nucleotide variants (SNVs) in the PIK3CA ^H1047R^ gene. In this design (Figure 2), RCA is amplified with the aid of the circular probes and the primers, mutant targets, and mixing with wild-type targets, with LODs up to 10 aM, thanks to signal amplification. The combination of CRISPR/Cas12a and RCA technology ensured the sensitivity and specificity for SNV detection [56]. 

Branched rolling circle amplification (BRCA) has been reported as superior to linear rolling amplification due to its exponential amplification power [57]. Ma et al. coupled the BRCA with pyrophosphate-sensitive fluorescence, generating terpyridine-Zn (II) complex as a reporter probe. A detection limit of 0.1 pM was reported in this design [57]. Li et al. developed a hyperbranched rolling circle amplification (HRCA)-based fluorescence biosensor for detection of SNPs associated with the therapy of chronic hepatitis B virus infection [58]. In addition, loop-mediated isothermal amplification (LAMP) has been applied widely to nucleic acid detection. Sun et al. reported a simple artificial mismatched ligatio (AML) probe combined with the ligase-assisted LAMP amplification (AML-LAMP)-based genotyping assay by combining the AML probe with the LAMP reaction for sensitive and superspecific genotyping of SNVs. With the detection limit of 10 aM, the sensor was capable of discerning up to 0.01% of mutated DNA [59], without high temperature and complex thermal cycling equipment.

**Figure 3:**
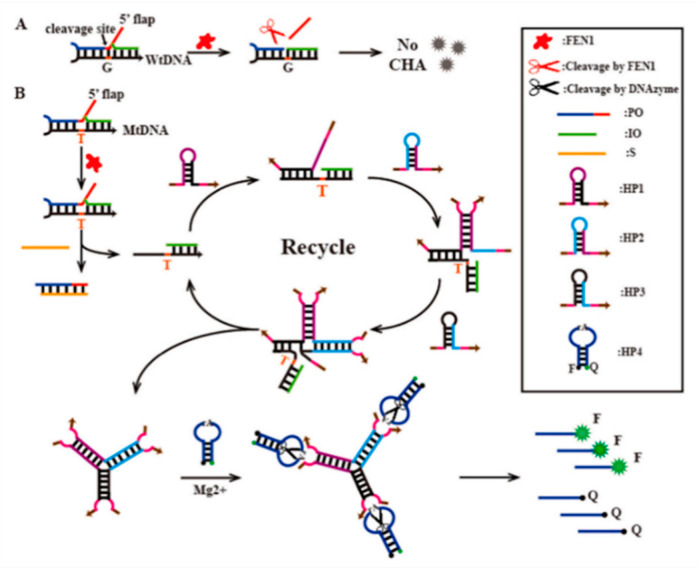
(**A**) Illustration of PO/IO/WtDNA strands hybridize to form trinucleotide repeat (TR) substrates that were recognized and cleaved by FEN1 and cannot trigger subsequent CHA reactions; (**B**) Schematic diagram of DNAzyme ligation triggered by CHA-induced invasion detection for ultrasensitive and specific detection of SNPs in the K-ras gene. Reproduced with permission from [63]. Copyright 2022 Elsevier.

To increase the discriminative ability of SNP detection, hairpins or other structural elements were added to the probe molecule to achieve highly selective single-base mutation detection [60,61,62]. Li and partners designed a tripartite DNAzyme ligation formation based on catalytic hairpin assembly (CHA) triggered by flap endonuclease 1 (FEN1) invasion detection for specific recognition of K-ras gene fragments (Figure 3). Hybridization of single-base mismatched DNA of the K-ras gene with sensing probes inhibited the enzymatic activity of FEN1, which triggered the subsequent CHA of the three hairpins, in turn, to form triplet ligation. In addition, fluorescence-quenched signal probes could be cleaved by the DNAzymes cycle to restore the enhanced fluorescence response. This detection strategy had outstanding specificity and high sensitivity, with the reported detection limit of 4.23 fM, in addition to its outstanding specificity, which was expected to become a powerful molecular tool for early cancer diagnosis and clinical research [63]. 

**Figure 4:**
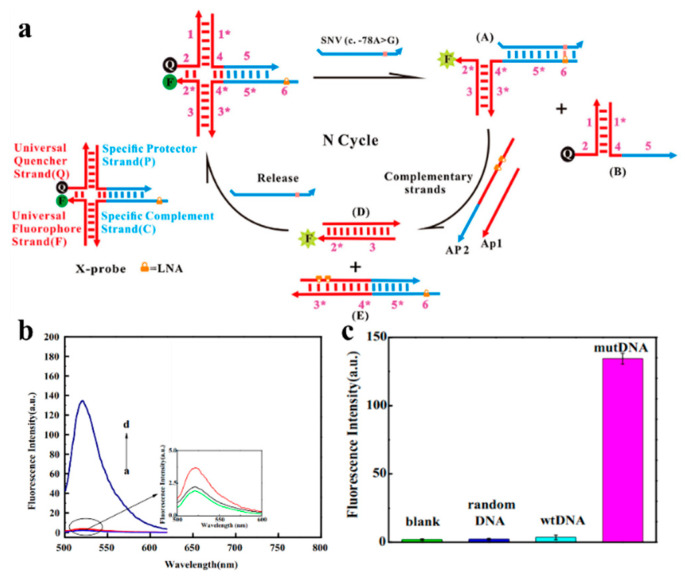
(**a**) Illustration of the universal LNA-integrated X-shaped DNA probes for the fluorescent biosensor; (**b**,**c**) fluorescence spectra of no mutDNA (background, curve a), random DNA (curve b), wtDNA (curve c), and mutDNA (curve d). Reproduced with permission from [64]. Copyright 2017 Elsevier.

Wu et al. developed a selective fluorescent biosensor based on an X-shaped probe, locked nucleic acid (LNA), and toehold-mediated strand-displacement reaction (TMSDR) (Figure 4). The LNA-integrated X-shaped probes could be isolated from target-specific regions and had prominent discernibility for β-thalassemia SNV. The introduction of the TMSDR-assisted recycling amplification system significantly improved the sensitivity. The detection limit under this strategy was up to 6 fM [64]. 

Compared with organic dyes and fluorescent proteins, nano-fluorescent materials have excellent photostability, high fluorescence quantum yield, and size-dependent optical properties. In addition, the large specific surface area of nanomaterials facilitates the covalent binding of various biorecognition molecules [65,66]. The integration of SNPs with fluorescent nanomaterials endows the biosensor with the remarkable feature of high sensitivity.

**Figure 5:**
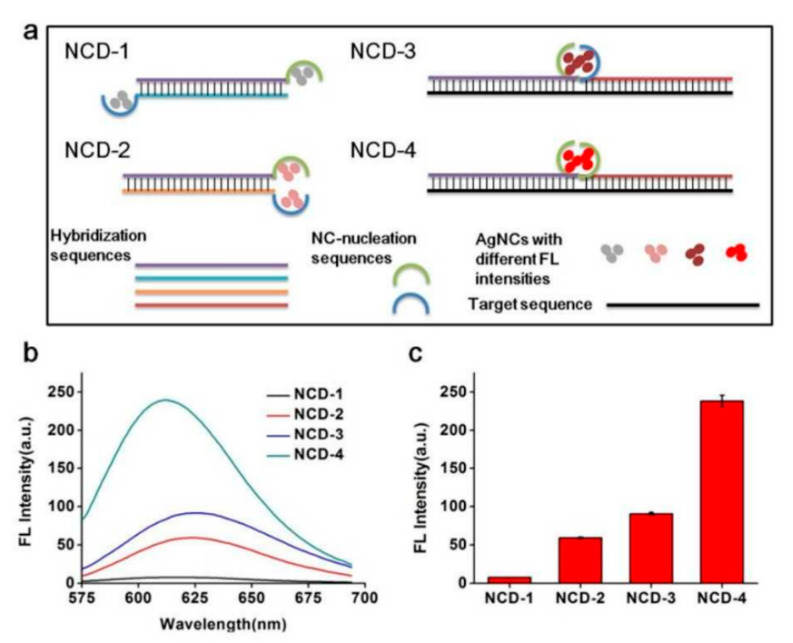
(**a**) Illustration of diversified hybridization structures of novel fluorescent molecular probes based on different nanocluster beacons; (**b**) Fluorescence spectra of NCD-1, NCD-2, NCD-3, and NCD-4 after hybridization; (**c**) Fluorescence intensities of NCD-1, NCD-2, NCD-3, and NCD-4 after hybridization at maximum emission. Reproduced with permission from [72]. Copyright 2017 Analytical Chemistry.

AgNCs become suitable fluorescent nanomaterials due to their high fluorescence efficiency, good biocompatibility, and excellent photostability [67,68,69,70,71]. Liu et al. developed a novel AgNCs-based fluorescent biosensor for SNP identification. They created a fluorescence mechanism based on AgNCs and were able to form nanocluster dimers (NCDs) (Figure 5). When the interactions between SNPs occurred at diverse positions, NCD increased the fluorescence intensity because of the spacing between the two AgNCs. As the mismatched base position in the target DNA gradually moved, the fluorescence intensity of NCD decreased proportionally. This technology uses nanocluster probes to precisely locate the positions of different SNPs in a sensitive, low-cost, and enzyme-free manner [72]. In addition, Guo et al. developed a DNA probe with an inserted cytosine loop as double-stranded scaffolds to generate fluorescent AgNCs. The generation of fluorescent AgNCs was highly sequence-dependent and could specifically identify single-nucleotide mutations located outside the two bases of the nanocluster formation site, the sickle cell anemia mutations [73]. For further applications of AgNCs in the detection of SNPs, Martinez and colleagues also reported a novel fluorescent molecular probe for a nanocluster beacon (NCB), which emitted different colors when bound to SNP targets. Depending on the recognition of AgNCs with DNA enhancer sequences, the fluorescence emission color of NCBs could transform significantly. This SNP assay has been varied in three synthetic DNA targets and six disease-associated SNP targets [74].

**Figure 6:**
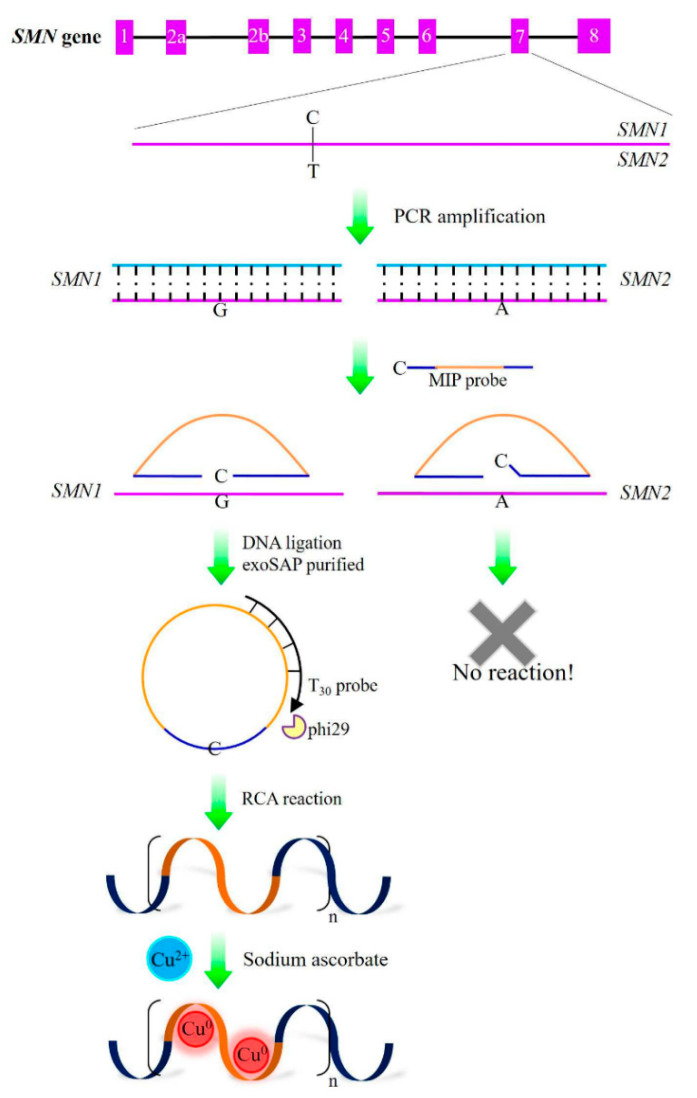
SMN genotype fluorescence detection based on MIP-RCA reaction with synthesis of poly T-templated CuNCs. Reproduced with permission from [78]. Copyright 2020 Analytica Chimica Acta.

The common fluorescent metal nanoclusters mainly include gold nanoclusters (AuNCs), silver nanoclusters (AgNCs), and copper nanoclusters (CuNCs). Due to their good photostability and biocompatibility, metal nanoclusters are widely used as fluorescent probes for chemical sensing and biological detection [75]. Among metal nanoclusters, CuNCs are considered to be very promising green nanomaterials due to their rapidly in situ production, low price, and non-toxicity. In addition, DNA-mediated fluorescent CuNCs synthesis with large Stoke shifts has great potential to diagnose nucleic acids in biosensor systems [76,77]. Recently, Chen et al. established a fluorescence detector for diagnosing spinal muscular atrophy (SMA) based on the poly-T-mediated CuNCs (Figure 6). They used molecular inverted probes to identify nucleotide variations in genes and perform roll-around amplification with primers to produce poly-T single-stranded DNA. The fluorescence of CuNCs was detected only existing in the SMN1 gene. This strategy was well adapted to a valid and specific method of 65 DNA samples in clinical trials [78]. In addition, Jia et al. reported that dsDNA-based copper nanoclusters (CuNCs) could identify mismatches in DNA sequences. For the dsDNA-templated CuNCs, the fluorescence intensity is closely related to the base type located in the groove. The results of this study provided sensitive and rapid fluorescence detection of the mismatch types in specific DNA sequences [79].

SNPs have been confirmed in quantum dots (QDs) in microarray format. The result showed that due to the large size of quantum dots, the surface density was lower, so that the sensitivity of the QD-labeled sensor was lower than that of fluorescent dye labeling [80]. In order to effectively distinguish between fully matched DNA and mismatched DNA, Guo and coworkers used streptavidin-coated quantum dots (strAV-QDs) to label fixed MBs to detect SNPs on target DNA sequences, which had an increased signal–noise ratio to 8, and the detection limit as low as 10 pM, exhibiting a genotype-dependent fluorescence signal [81]. 

Genetic testing in clinical practices demands efficient screening methods that meet the requirements of point-of-care testing (POCT) strategies. On this ground, different detection platforms with simple architecture and less expensive instruments have been developed [82,83]. Watterson et al. developed a disposable fiber-optic biosensor for SNP detection associated with SMA. The system used total internal reflection fluorescence (TIRF) to identify motifs capable of distinguishing PCR specimens of 202 base pairs acquired from patients. Real-time and significant discrimination can be performed with widely varying ionic strengths, significantly reducing reaction time and enabling evaluation to be completed in less than 1 min [84,85]. 

For a comparison, Table 1 summarizes the fluorescent strategies used for SNP detection.

### 2.2. Electrochemical Biosensors

Biosensing methods based on electrochemical transduction mechanisms have been reported to be sensitive, selective, rapid, and amenable to miniaturization and experimental convenience [89,90,91,92,93,94]. A variety of strategies aiming at improving the target recognition and signal transduction performance have been developed [95,96,97,98,99]. Owing to the features of enzyme-free, LNA-integrated, and toehold-mediated SDR techniques, Gao et al. developed a reusable DNA sensor for SNP detection. This biosensor not only offered specific discrimination for SNP detection, but also was able to function even in contaminant-ridden samples, such as human urine, soil, saliva, and beer [100]. 

**Figure 7:**
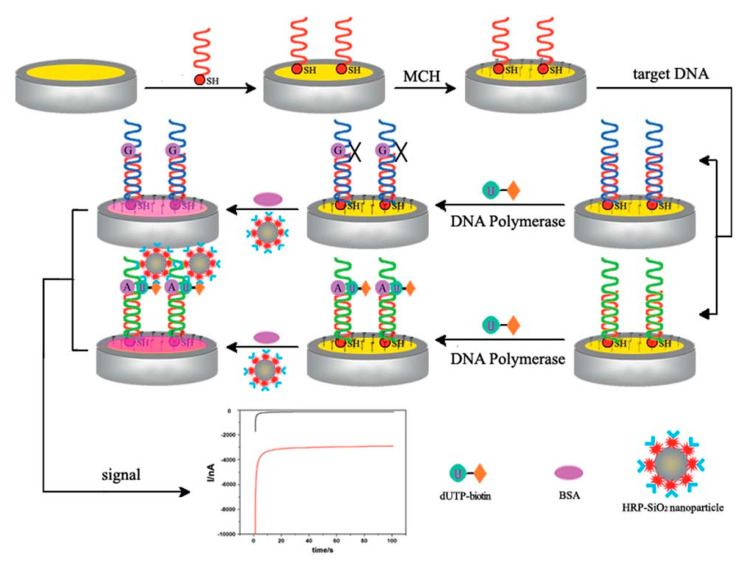
Illustration of the proposed electrochemical biosensor for detecting SNPs in the K-ras gene. Reproduced with permission from [101]. Copyright 2016 Royal Society of Chemistry.

Zhao and colleagues developed an ultrasensitive electrochemical method to detect point mutations in the K-ras gene by combining streptavidin horseradish peroxidase (streptavidin-HRP)-modified SiO_2_ nanoparticles and DNA polymerase in the sandwich design. In this design (Figure 7), the streptavidin-HRP-SiO_2_ nanoparticles had the effect of amplifying the signal. HRP reacted catalytically with 3,3’,5,5’-tetramethylbenzidine (TMB) to produce an electrochemical signal. A wide linear range (0.001–100 pM) and 0.42 fM detection limit was reported under this design [101]. Its simplicity and cost-effectiveness give it an advantage over PCR-based assays. 

To solve the problem of interference by trace mutants and endogenous substances in actual samples, Liu et al. proposed a unique DNA point mutation detection strategy based on the ligase chain reaction (eLCR) of novel electrochemical biosensors. In this design, a porous monolayer was constructed by modifying bovine serum albumin (BSA) molecules on gold electrodes, which relied on Au-S bonds to link double-stranded DNA generated by LCR. This method identified mutations in the *CYP2C19* gene (G681A) with remarkable specificity and sensitivity, without the involvement of pre-PCR. The detection limit of this sensing method was 0.5 fM. Due to its advantages of simple primer design, easy handling, and easy miniaturization, it has potential applications in clinical analysis and genetic diagnosis [102].

**Figure 8:**
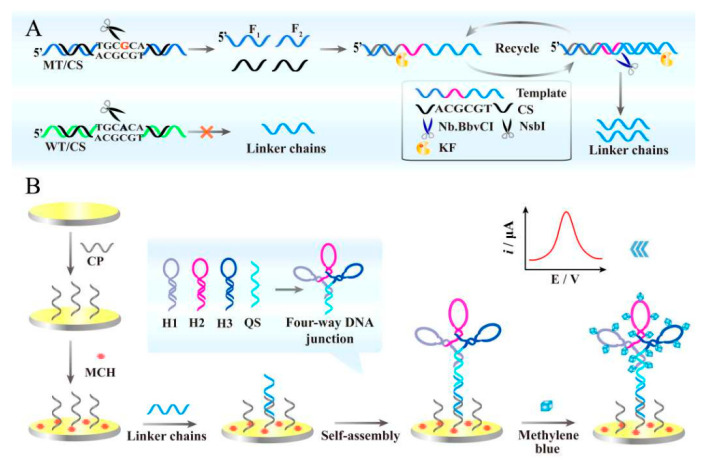
(**A**) Illustration of the properly matched MT/CS dsDNA was recognized by NsbI and cleaved to initiate the SDA reaction; (**B**) Schematic diagram of the electrochemical DNA sensor based on NsbI-SDA and four-way DNA junction. Reproduced with permission from [103]. Copyright 2020 Elsevier.

PIK3CA gene mutation is one of the most common mutated types in human cancers, and its presence is often associated with low survival in patients. Wang and colleagues proposed an original electrochemical sensor for specific and ultrasensitive detection of mutations in the PIK3CA^H1047R^ gene, based on NsbI-restricted endonuclease-mediated strand displacement amplification (NsbI-SDA) and four-way DNA ligation to enhance the electrochemical response (Figure 8). It achieved ultra-sensitive detection by embedding methylene blue (MB) electroactive molecules in four-way DNA ligation to form a sandwich structure. With a detection limit of 0.001%, this biosensing method can be used to analyze mutated genes incorporated into human serum samples, demonstrating promising use in sensing analysis and clinical applications [103].

**Figure 9:**
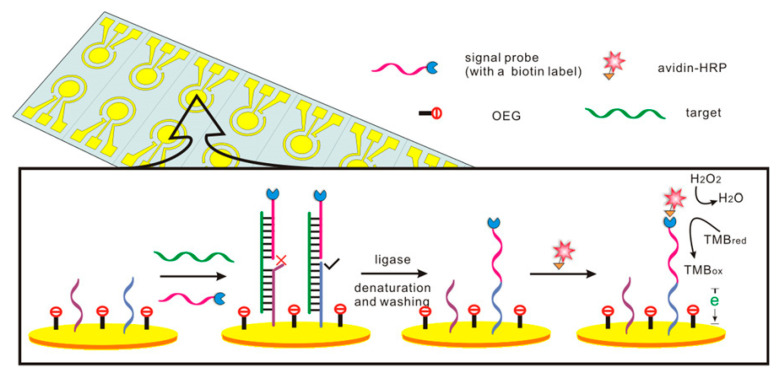
Electrochemical biosensor based on the ligation and ONS engineering for SNP detection method. Reproduced with permission from [104]. Copyright 2013 Elsevier.

Liu et al. proposed an oligonucleotide-incorporated non-fouling surface (ONS) to avoid nonspecific absorption (Figure 9). Using a sixteen-electrode array, they constructed a novel electrochemical biosensor capable of high-speed SNP testing at C680T and G681A in the human CYP2C19 gene. Capture probes with alternative terminal bases at the 3’-terminus were designed on the electrode surface. Only complete hybridization can ligate the two probes. A current signal sixteen times larger than the blank sample could discriminate ten percent of the single-base mismatch sequence [104]. Wan et al. also used a typical “sandwich” scheme and ONS engineering strategies to detect SNPs on the surface of gold electrodes. The ligation product can be catalyzed by peroxidase into an electrical signal. The approach to identify only a single-base mismatch could practically distinguish SNPs [105].

Notably, DNA sequencing and DNA microarrays are currently the most frequently used SNP identification means due to their high throughput and cost-effectiveness. Nevertheless, highly specific SNP detection is required, and nanotechnology-based methods can provide a solution [28,106,107]. Nanotechnology-enhanced electrochemical sensors show great potential in detecting mismatched base pairs in DNA [108]. 

**Figure 10:**
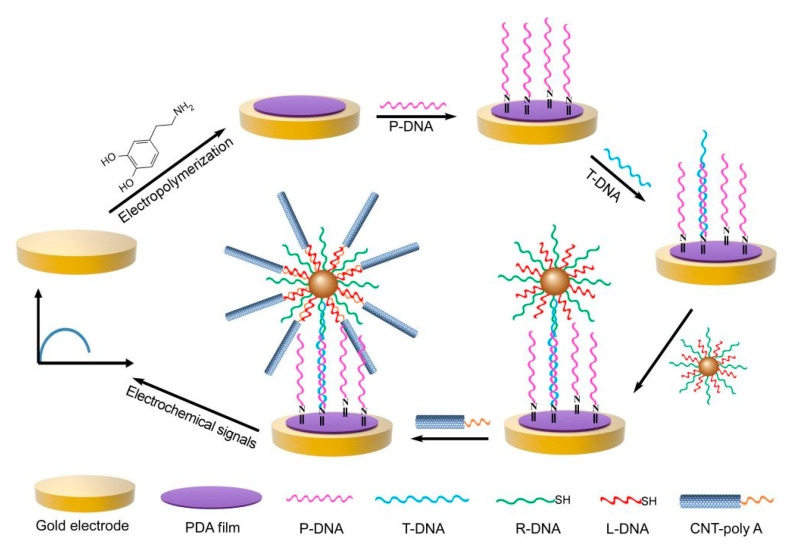
Schematic diagram of the process used by the electrochemical DNA biosensor to detect target DNA. Reproduced with permission from [114]. Copyright 2020 American Chemical Society.

DNA-stabilized gold nanoparticles (AuNPs) are widely used for SNP detection [109,110,111,112,113]. Han et al. developed a facile, ultrasensitive DNA biosensor based on urchin-like carbon nanotube–AuNP-conjugated (CNT-AuNP) nanocluster signal amplification. When the dopamine-modifying gold electrode was attached to the DNA probes, DNA-functionalized AuNPs were led to the biosensor through DNA bases complementation. Then, CNTs with end-modified DNA were linked with AuNPs to form 3D radial nanoclusters, which generated significant electrochemical signals (Figure 10). Due to the large contact surface area and ultra-strong electronic conductivity of the CNT-AuNP clusters, this 3D radial nanostructure exhibited ultrasensitive detection ability, good selectivity, and excellent stability and regeneration ability for DNA detection, which obtained a low LOD of 5.2 fM [114].

Moreover, nanotechnology-enhanced electrochemical biosensors have shown good prospects for distinguishing single-base mutations [108]. The application of graphene (GR) with a proverbial two-dimensional structure in electrochemical biosensors has aroused great interest due to its superior performance, including large surface area, easy electron transportation, and good biocompatibility [115]. Khoshfetrat & Mehrgardi developed a graphene–gold nanoparticle (GR-AuNPs) nanocomposite-based biosensor with a triple amplification strategy for SNP detection. This novel design exhibited outstanding sensitivity and specificity for G-T and A-C mismatch targets, with detection limits of 2 pM and 10 pM, respectively, and this GR-based assay could play a significant role in SNP detection for related diseases [116].

Hwang and partners developed a sensing platform for SNP label-free recognition combining DNA nanotweezer probes with GR field-effect transistor chips to improve analytical efficiency. This super-sensitivity assay demonstrated the ability to wirelessly transmit SNP detection-induced electrical signals in real time. DNA nanotweezers were fixed on the GR surface, and SNP genotyping was performed using the GR field-effect transistor sensor. Compared to previous studies, DNA nanotweezer probes increased sensitivity by more than 1000 times, significantly enhancing the analytical characteristics of SNP genotyping [117].

For a comparison, Table 2 summarizes the electrochemical strategies used for SNP detection. 

### 2.3. Other Biosensors

As mentioned above, fluorescent SNP biosensors have the advantages of high sensitivity, high selectivity, and high throughput. However, they are also very sensitive to some of the disturbances that typically occur in fluorescence measurements, such as background fluorescence and quenching effects. In addition, the measuring equipment is usually expensive, which increases the cost of fluorescent SNP detections. For electrochemical SNP biosensors, the electrodes can provide a platform for subsequent modification of various materials, with the aim of improving the sensitivity, selectivity, and stability. Electrochemical SNP biosensors have the advantages of low cost, fast response, high sensitivity, and easy to miniaturize, but the modification of electrodes is relatively complicated, and the stability of the recognition elements and detection repeatability is expected to be improved.

Besides fluorescent detection, other optical methods, such as colorimetric analysis [125], surface plasmon resonance (SPR) [126], and surface-enhanced Raman spectroscopy (SERS) [127], have been also used for SNP detection. 

Colorimetric assay, a technique for the determination of biological elements in solution with chromogenic reagents, has the advantages of simple operation, visibleness by the naked eye, and no requirement of expensive or complex instruments. In recent years, many colorimetric analysis strategies for mutation detection have been developed. Chen et al. developed a single-step, enzyme-free, non-labeled, universal strategy for the colorimetric detection of SNPs based on the G-quadruplex-mediated conversion of a colorless 2,2′-azinobis (3-ethylbenzothiozoline)-6-sulfonic acid (ABTS2-) to a green ABTS as the reporting signal for the presence of SNPs [128]. Wu et al. developed a simple and rapid colorimetric platform for amplified single base-pair mismatch detection based on the aggregation of exonuclease-sheared AuNPs. When the AuNP-binding probe binds to a perfectly matched target, the exonuclease activity of Exo III facilitates the target recovery process, which rapidly cleaves the DNA probe from the particle, producing an AuNP aggregation-induced color change. This change does not occur with DNA targets that contain single-base mismatches. This platform employs an AuNP-based, exonuclease III (Exo III)-amplified strategy to achieve colorimetric SNP detection at low nanomolar target concentrations [128,129]. Deng et al. developed a simple colorimetric assay for highly sensitive and specific detection of SNPs based on the separation of magnetic beads and the specificity of a mismatch-specific CEL II enzyme (surveyors nuclease) in cleaving mismatched (interfering) DNA duplexes to the excellent signal amplification power of DNAzyme. A detection limit as low as 0.40 fM and a dynamic range from 1.0 to 200 fM were reported [130]. 

The use of surface-enhanced Raman scattering (SERS) has increased significantly in the biomedical field. SERS is a powerful surface-sensitive method that relies on Raman signal resonance caused by molecular interactions with nanostructures or rough metal surfaces. SERS has shown great potential in the detection of unlabeled DNA. Compared with common biological methods (such as PCR), the DNA detection has advantages of sensitivity, specificity, and detection speed [131,132,133,134]. Ngo et al. developed a highly sensitive nanoplatform for DNA detection and SNP discrimination based on ultrabright SERS nanorattles and magnetic beads for malaria diagnostics. Under this strategy, a detection limit of approximately 100 attomoles was reported [135]. Lowe et al. proposed a multiplex SNP genotyping technique based on the ligase detection reaction (LDR)-SERS. In this platform, the diagnostic peak of Raman spectra was clearer than that of fluorescence spectra, which allowed the technology to improve the reusability of current homogeneous detection maps by preventing spectral overlap. The SERS signal acquired the LOD of 10 pM [136].

Surface plasmon resonance (SPR) is also widely known in optical biosensor strategies, especially in SNP detection [137,138]. SPR technology is based on the detection of refractive index changes due to molecular interactions on metal surfaces or other conductive materials by surface plasma waves [139]. Recently, Yi et al. reported a SPR method for apoE gene and genotype discrimination associated with Alzheimer’s disease (AD). Due to complete complementarity with the pre-immobilized biotinylated probes, the HhaI enzyme selectively cleaved GCGC base pairs in the duplex, whereas the digestion reaction was prevented in the presence of the single-base mismatch (GTGC). The detection level of 50 fM was acquired [140]. Due to the capacity for multiplexed analysis, surface plasmon resonance imaging (SPRi) biosensors have been widely used for the assay of SNPs [141]. Using the Au nanoparticle tag, SPRi increased the detection sensitivity of target oligonucleotides by more than 1000 times, with a LOD of 10 pM [142]. Li and coworkers combined the surface enzymatic ligation reaction and enhanced hybridization adsorption of gold nanoparticles on DNA microarrays. In this strategy, the detection limit of SNPs in the BRCA1 gene associated with breast cancer by SPRi was 1 pM [143]. 

**Figure 11:**
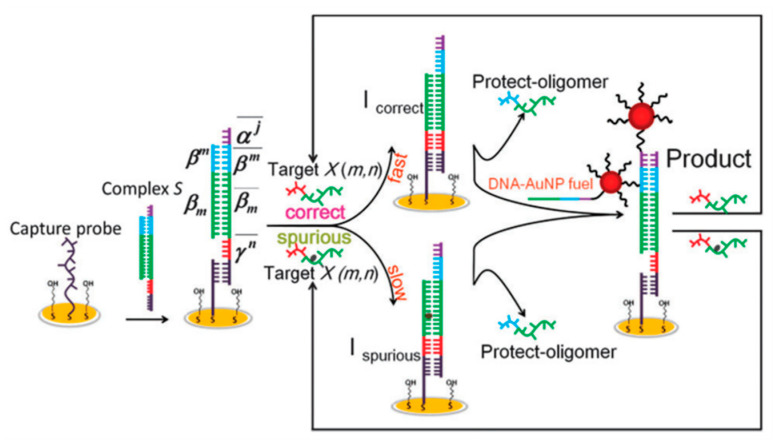
Schematic diagram of single-base mutation detection based on QCM strategy. Reproduced with permission from [154]. Copyright 2015 Royal Society of Chemistry.

The QCM biosensor is an extremely sensitive mass sensor capable of measuring subnanogram levels of mass changes [144,145,146,147]. QCM biosensors are suitable for direct and label-free monitoring of affinity interactions of biomolecules [148,149,150,151]. In the efforts to develop a new strategy for simple, selective, and sensitive detection of SNPs, different designs including TSDR and nanomaterials-based QCM detection platforms have been reported [152,153,154,155]. Compared with classical sandwich hybridization, toehold-mediated DNA assembly has significant advantages. Li et al. developed a QCM sensor, driven using toehold-mediated and DNA-AuNPs probes, for the detection of single-base mutations (Figure 11). DNA-AuNPs can release the target sequence back into solution; therefore, cyclic initiation of the strand displacement reaction can be achieved by displacing the target sequence from the linker oligomers. This design helped to improve the sensitivity. Thus, QCM-based DNA-AuNP probe-driven strand displacement reactions enable clear discrimination of single-base mismatches. The detection limit was 35 pM [154].

## 3. Conclusions

SNPs have attracted extensive attention in the field of genetics because of their effect on DNA sequence polymorphisms caused by single-nucleotide variation. Researchers can use these SNPs to obtain a wealth of molecular pathological information, early screening of disease, and assessment of the loss of heterozygosity for genetic testing. Over the past decades, researchers have been working to develop sensitive, fast, convenient, and cost-effective SNP detection methods. Biosensing technology solves many problems in mutation detection, including multi-sample detection, low work efficiency, and difficulty detecting SNPs in a dsDNA, as well as sensitivity, selectivity, and accuracy. Assays are selected based on individual needs, available materials, and mutations of interest. Despite significant advances in biosensing detection methods, more novel mutation detection strategies are needed to assess these issues for timely diagnosis and accurate detection of drugs and diseases, which will greatly facilitate the clinical application.

Nowadays, sensing technologies have become more dynamic, powerful, and versatile. Significant progress has been made in designing biodiagnostic tools to detect low-abundance SNPs. A variety of assay strategies, such as fluorescent, electrochemical, mass, and other optical biosensors, have been developed for SNP detection. In the design of SNP biosensors, the combination of nanomaterials and amplification strategies is promising in the enhancement of detection sensitivity and specificity, helping to facilitate the universal application of SNP assessment. The introduction of biologically active substances and immobilized materials to improve detection capabilities has yielded some interesting results. The use of enhanced substrates, such as graphene, metal nanoclusters, quantum dots, and core-shell nanomaterials, could significantly improve the sensitivity and selectivity of SNP biosensors. DNA amplification techniques, such as RCA, SDA, and LCR, have made significant contributions to the sensitivity improvement of SNP analysis techniques. To achieve high specificity, which is essential and difficult in SNP detection, technologies such as CRISPR/Cas and structured probes, as well as mismatch-specific enzymes and proteins, are helpful to improve detection selectivity. Therefore, future research will continue to explore various techniques to address the above challenges and the designing and manufacturing of biosensors with high throughput, higher sensitivity, and higher specificity to facilitate the general applicability of SNP detection.

## Figures and Tables

**Table 1:** Fluorescent biosensors with various signal amplification strategies for mutation detection.

Signal Amplification Strategies	Target Mutation	LOD	Ref.
CRISPR/Cas12a with RCA	SNV of the PIK3CA ^H1047R^	10 aM	[56]
Invader assay-induced multiDNAzyme junctions	SNP	4.23 fM	[63]
Universal locked nucleic acid-integrated X-shaped probe	SNP	6 fM	[64]
Core-shell gold nanocube (AuNC) and plasmon-enhanced fluorescence (PEF)	SNP	1.3 pM	[86]
RT-PCR associated with G-quadruplex RCA	Multiple SNPs	8.3 fg	[87]
Fluorescence polarization (FP) and target-initiated rolling circle amplification (RCA)	KRAS G13D and G12D mutations	5.88 pM	[88]
Multiple primers-mediated RCA coupled with a graphene oxide-based fluorescence	Multiple SNPs	0.87 fM	[53]

**Table 2:** Electrochemical biosensors with diverse signal amplification assays for mutation detection.

Signal Transduction	Biosensor Platform	Target Mutation	LOD	Ref.
Amperometric	Electrochemical ligase chain reaction (eLCR)	CYP2C19 (G681A) in human whole-blood samples	0.5 fM	[102]
Impedimetric	Graphene	Apo E gene	G-SL: 50 nM G-FL: 6.6 pM	[118]
Impedimetric	Anchor-like DNA (alDNA) electrochemical biosensor	KRAS G12D mutation	0.1 pM/100 pM (total/mutant DNA)	[119]
Amperometric	PNA/ds-DNA triplex formation	p53 gene	10^−6^ M	[120]
Voltammetric	MDB as a hybridization indicator	VDR gene	10.9 pmol/100 mL	[121]
Voltammetric	HCR and SDR	P53 gene	20 aM	[122]
Impedimetric	CRISPR/dCas9-powered impedimetric	ctDNA, PIK3CA exon 9 mutation	0.65 nM	[123]
Voltammetric	CRISPR/cas-enhanced electrochemical biosensor	SNPs	10 fM	[124]

## Data Availability

Not applicable.
